# Memoir on the Sex of the Child as a Cause of Difficulty and Danger in Human Parturition

**Published:** 1845-04

**Authors:** 


### ART. XV.

*Memoir on the Sex of the Child as a Cause of Difficulty and Danger in Human Parturition.* By J. Y. SIMPSON, M.D. (*Reprinted from the Edinburgh Medical and Surgical Journal for Oct.* 1844.) pp. 55.

IT is a well known fact that the larger proportion of still-born children are males; and another equally well substantiated, which was first mentioned by the late Dr. J. Clarke, is that the dimensions of the head, in the male fetus, are, on the average, considerably greater than in the female. These two facts form the groundwork of Dr. Simpson's essay, in which he endeavours to prove, "That the sex of the child has a very marked and demonstrable influence upon the difficulties and dangers of human parturition, in reference to the fate both of the mother and of the infant."

The elaborate tables contained in Dr. Collins's 'Treatise on Midwifery,' furnish Dr. Simpson with the data whence he deduces the following propositions:

1st. That the dangers and difficulties of parturition are greater to the mother in male than in female births.

2d. That the dangers and accidents from parturition and its results are greater to the child in male than in female births.

3d. That for the very marked difference between the difficulties and perils both to the child and the mother in male from that which exists in female births, there is no other traceable cause in the mechanism of parturition, except the larger size of the head of the male child.

His first proposition is established by showing, that in labours presenting morbid complications and difficulties, the child is much oftener male than female; and further, that the larger number of women who die of parturition, or its immediate consequences, have given birth to male children.

The former of these facts is well exhibited in the following table.

| " Nature of complication. | Total cases. | No. of Male children. | No. of Female children. | Proportion of Males to Females, as |
|---|---|---|---|---|
| Tedious labours . | 109 | 65 | 54 | 148 to 100 |
| Convulsions . . | 28 | 17 | 11 | 153 ,, |
| Puerperal fever . | 88 | 54 | 34 | 161 , |
| Rupture of uterus . | 34 | 23 | 11 | 207 |
| Post-partum hemorrhage | 44 | 31 | 13 | 240 |
| Forceps cases . | 24 | 16 | 8 | 200 ,, |
| Crotchet cases . | 74 | 50 | 24 | 208 ,, |
| Total . . | 401 | 256 | 155 | 165 to 100" |

(p. 7.)

The results afforded by this and by the rest of the tables require a slight correction, which consists in deducting 6 per cent. from the column of male children, that being the excess of male over female births in the Dublin Lying-in Hospital.

The number of deaths of mothers is too small perhaps to warrant any very positive conclusions; though when we find that in 105 instances the women had given birth to male children, and in 49 only to female, the difference certainly appears too considerable to be the result merely of accident.

A very striking series of facts is adduced by Dr. Simpson in support of his second proposition. During Dr. Collins's superintendence of the Dublin Lying-in Hospital, 1121 children were still-born; of whom 614 were males, 507 females, or the males were to the females in the proportion of 122 to 100. It might indeed be thought that this disparity is in part owing to a larger number of male than of female children dying before the commencement of labour. This, however, does not appear to be the case, since of the 527 children who were putrid before birth, 257 were males, and 270 females. A calculation founded on the remaining 594 children gives, as the result, 357 males to 237 females: or, in other words, the number of male children who died during the actual process of parturition was to that of females as 151 to 100.

The evidence adduced in proof of the assertion, that, of children born alive, more males than females suffer from the morbid states and injuries resulting from parturition is too meagre to be of much value. Dr. Simpson's next statement is to the effect that more male than female children die in the earliest periods of infancy, and that this disproportion between the mortality of the two sexes gradually diminishes from birth onward. This fact was first noticed by M. Quetelet, in his 'Recherches sur la Reproduction et la Mortalité de l'Homme;' and, according to him, "the ratio of deaths of the sexes during the first two months after birth is as 4 to 3; during the third, fourth, and fifth months, as 5 to 4; and after the eighth or tenth month, scarcely any difference exists. The table Dr. Simpson has compiled from Dr. Collins's work shows that 80 males and 59 females died during the first week, or that the proportion of male to female deaths was 136 to 100. The mortality tables of the Registrar-General yield nearly the same results for the first month of existence, since they show that 137 males died during that period for every 100 females.

Having thus established his first two propositions, Dr. Simpson proceeds to the third. On the fact that, of the 527 still-born putrid children, 257 were male and 270 female, he grounds the assertion that, of the children who die in utero before the commencement of labour, as large a proportion are female as male. Any assertion with reference to this subject that rests on a small number of figures must necessarily be looked on with suspicion. The opinion generally entertained is, that a greater mortality prevails before birth among male than among female infants, but we are not aware of the existence of any conclusive evidence on this point. The only data, indeed, that we know of besides what Dr. Collins's tables furnish are derived from the bills of mortality of the city of Hamburgh.* According to them, the proportion of the sexes in cases of premature still-born children is $52\frac{1}{3}$ males to $47\frac{2}{3}$ females.

Dr. Simpson next adduces illustrations of the greater size and weight of the male than of the female fetus, and then details the observations of the late Dr. Clarke, which established the fact that the head of the male fetus, measured across from ear to ear, is nearly $\frac{2}{8}$ of an inch greater than that of the female, $\frac{3}{8}$ of an inch greater in circumference, and nearly $\frac{1}{8}$ of an inch in the transverse diameter. Dr. Simpson, drawing from these facts a somewhat wider inference than Dr. Clarke had done, attributes

* Busch und Moser Handwörterbuch der Geburtskunde, Bd. i, p. 29.

to this excess in the dimensions of the male head the greater number of complications and casualties accompanying the birth of males. His statement, however, that no other traceable cause for this difference in the mortality of the two sexes is to be found in the mechanism of parturition, is not strictly accurate. Preternatural presentations are considerably more frequent in the case of male than of female children; a circumstance which, wholly independent of the larger size of the male fetus, would account for a certain excess of suffering to the mother and of mortality to the infant. This excess was estimated by Riecke to be in the proportion of 1·40 to 1; and of the 409 preternatural presentations recorded in Dr. Collins's work, 218 occurred in the case of male children; being in the proportion of 114 to 100; or, applying to it the same correction as is employed by Dr. Simpson, 108 to 100.* It must remain for more extended researches to show how far this excess of preternatural presentations in the case of such infants is real; we wish only to prove that this part of the case does not at present admit of being stated quite so absolutely as Dr. Simpson has done.

He next proceeds to show that the excess of male infants increases in direct proportion to the difficulty of the labour; a fact very well illustrated by the following table:

| "Nature of the labour and complication. | Total cases. | No. of Male children. | No. of Female children. | Proportion of Males to Females. | Ratio of excess of Males. |
|---|---|---|---|---|---|
| Labours generally | 16654 | 8548 | 8069 | 106 to 100 | 6 |
| Tedious labours | 109 | 65 | 44 | 148 ,, | 48 |
| Forceps cases . | 24 | 16 | 8 | 200 ,, | 100 |
| Crotchet cases . | 74 | 50 | 24 | 208 ,, | 108" |

(p. 27.)

The same fact had already been exhibited, in a somewhat different manner, by Dr. Clarke, who showed that, in cases of head-presentation, the excess of males among still-born children increases in direct proportion to the difficulty and protraction of the labours. Very powerful indirect proof of this is afforded by another table:

| "Non-putrid children stillborn. | Total. | Males. | Females. | Proportion of Males to Females. |
|---|---|---|---|---|
| At full time . . | 532 | 320 | 212 | 151 to 100 |
| Prematurely . . | 62 | 34 | 28 | 121 to 100" |

(p. 30.)

Which proves that among premature children whose head is smaller, and its passage through the pelvis consequently attended with less difficulty, the proportion of still-born males to still-born females is by no means so great as in the case of children born at the full time.

* We observe that Dr. Simpson quotes this fact from a notice of Riecke's work in the Archives Générales de Médecine, where the French writer has represented his statement as referring to artificial labours of all kinds. Our authority is Burdach, who in his Physiologie, Bd. iii, Seite 84, represents Riecke's statement to refer to those cases of labour in which the necessity for manual interference arose from preternatural presentation of the child.

The occurrence of greater dangers, and of more deaths, both of mothers and infants, in first labours than in subsequent ones, does not appear to us to have been adduced in evidence on this point with propriety; since the grand impediment to the passage of the head—that presented by the pelvis—must remain the same in subsequent labours; and the chief causes of difficulty peculiar to first labours depend, for the most part, upon dynamic causes on the part of the mother.

Dr. Simpson's opinions, however, do not rest on mere indirect evidence, or on arguments deduced from any supposed effect of the larger size of the head in the male fetus, but he proves that the average duration of labour is longer with male than with female children; and, further, that the difference in this respect becomes greater in proportion to the severity and dangerous character of the labour. This fact is partly shown by 427 observations made at the Edinburgh Lying-in Hospital, which yield an average duration of labour of eleven hours thirty-eight minutes in the case of male children, and of nine hours thirty-four minutes in the case of female children, which gives an excess of two hours and four minutes in the duration of labour with male children. Data furnished by Dr. Collins's work support the second part of this statement, as is seen by the following table:

| " Labours. | The average duration of the male births was | | The average duration of the female births was | | The average greater length of the male birth was | |
|---|---|---|---|---|---|---|
| | Hours | Minutes | Hours | Minutes | Hours | Minutes |
| With stillbirth (310 males and 197 females) | 14 | 27 | 12 | 10 | 2 | 17 |
| Morbidly tedious (56 males and 41 females) | 43 | 18 | 40 | 12 | 3 | 6 |
| Requiring crotchet (44 males and 24 females) | 42 | 22 | 38 | 20 | 4 | 2 |
| Leading to death of the mother (86 males and 44 females) . . | 18 | 9 | 12 | 58 | 5 | 11" |

(p. 44.)

We have now noticed the whole of the evidence adduced by Dr. Simpson in proof of the influence of the size of the head of the male fetus in causing greater difficulty in the labour and a higher mortality, both of mothers and children. He adds some remarks on the peculiarly tender structure of the brain in infancy, and on its larger size as affording an explanation of the serious results that may follow the pressure of the head in the pelvis; and concludes with some observations on "the extent of influence exerted by the male sex of the infant upon the general maternal and infantile mortality during parturition, and for some time subsequent to it." The conclusions at which he arrives are certainly startling. We give them in his own words:

"In the returns of Drs. Clarke and Collins, we have reports in the Dublin Hospital of the sex of the child in 368 cases in which the mother died from labour or its consequences. In 231 instances the child was male, in 137 cases it was of the female sex. The proportion of maternal deaths after male and female births, was therefore as follows:

| Total Deaths. | With male children. | With female children. | Proportion of males to females. |
|---|---|---|---|
| 368 | 231 | 137 | 168 to 100 |

"If we venture, then, to compute from these data that, out of 250 mothers that die in childbirth from parturition or its effects, 150 have given birth to males, and 100 to females, as the above table would seem to show, then we have in every 250 natural deaths an *actual excess* of 50 cases of loss of mothers after male births, for which (for reasons which we have previously stated at length) we can find no other explanation than the greater comparative size of the male head, and we have already attempted to prove that this explanation is in itself logically and amply sufficient to account for the consequences which we have attributed to it. In other words, among every 100 parturient mothers that die, we have 40 perishing after producing female children, other 40 perishing after producing male children, and the remaining 20 in the 100 perishing also after the birth of males; but so far forming a regular and constant *excess* of 20 per cent. of deaths in connexion with male births, traceable to no other cause than the sex and the consequent size of the infant. One in every five maternal deaths, or 20 in every 100, and 200 in every 1000, are so far the direct or indirect consequences of the greater dimensions of the head of the male infant. Now, at the present day, in Great Britain, upwards of 3000 women die every year from childbirth or its immediate effects. Hence, according to the above computation, 600 of these 3000 cases, as forming the *excess* of male over female births, are more or less immediately the results of the sex and size of the male infant. In Great Britain, therefore, the valuable lives of 500 mothers (to speak within the terms,) are lost in childbirth through the influence and agency of the cause in question.............

"We have seen, under our fourth proposition, that in the Dublin Hospital there died, during the process of parturition, and probably as a consequence of the injuries to which they were subjected, 151 male children for every 100 females. According to the mode of argument followed in the preceding paragraph, there was thus an *excess* of 50 male deaths among every 250 children, or 20 in every 100, referrible to the greater size of the head of the male infant. Further, we may take it for granted that, on a low computation, 1 in every 50 children born dies during labour, about 1 in every 25 cases being a still-birth. To be certain, however, not to overstep our limits, let us reckon only one in every 75 children to die during parturition, and 1 in every 5, or 20 per cent., of those that thus perish to be formed by that excess of mortality of males over females which we can trace to no other cause than the influence of the greater dimensions of the male head. In England and Wales about 500,000 births take place annually. By the above computation, more than 6500 of the offspring of these births die during labour, and one fifth of that number are lost in consequence of the sex and size of the male child. In Great Britain, therefore, the lives of 15,000 infants are annually lost in childbirth from the operation of this agency." (pp. 51-2.)

Applying a similar mode of reasoning to account for the excess of male infants who die within the first year after birth, he concludes "that there die annually in Great Britain upwards of 5000 children within the first year after birth, whose death is referrible to the influence of the sex and greater size of the male head during labour." (p. 53.)

We have noticed this pamphlet at much greater length than we usually do such short essays, on account of the very interesting results at which Dr. Simpson has arrived. The fact that a disproportion exists between the size of the head of male and female infants has been known to the profession ever since the time of Dr. Clarke, and that disproportion has been regarded as a cause of greater protraction of the labour and of a greater number of still-births in the case of male children. Dr. Simpson cannot therefore be considered to have discovered any new fact; but he has done what is scarcely less valuable, in showing the great importance of the fact, and the extent to which it explains that problem of which no solution has hitherto been offered, as to the cause of the great excess of male mortality soon after birth. We think, indeed, that the numbers with which Dr. Simpson works are too small for us to feel assured that the results they yield are thoroughly accurate. We need, too, more information than we at present possess as to the sex of abortions, and also as to the comparative frequency of preternatural presentations in the two sexes before we can give a thorough assent to some of Dr. Simpson's statements. Still we have no doubt but that the main facts will be substantiated by more extended observations: and we consider the essay to be an exceedingly valuable contribution to midwifery statistics.

---

